# Host Responses to Group A Streptococcus: Cell Death and Inflammation

**DOI:** 10.1371/journal.ppat.1004266

**Published:** 2014-08-28

**Authors:** James A. Tsatsaronis, Mark J. Walker, Martina L. Sanderson-Smith

**Affiliations:** 1 Illawarra Health and Medical Research Institute (IHMRI), School of Biological Sciences, University of Wollongong, Wollongong, New South Wales, Australia; 2 Australian Infectious Diseases Research Centre, School of Chemistry and Molecular Biosciences, University of Queensland, St. Lucia, Queensland, Australia; International Centre for Genetic Engineering and Biotechnology, India

## Abstract

Infections caused by group A *Streptococcus* (GAS) are characterized by robust inflammatory responses and can rapidly lead to life-threatening disease manifestations. However, host mechanisms that respond to GAS, which may influence disease pathology, are understudied. Recent works indicate that GAS infection is recognized by multiple extracellular and intracellular receptors and activates cell signalling via discrete pathways. Host leukocyte receptor binding to GAS-derived products mediates release of inflammatory mediators associated with severe GAS disease. GAS induces divergent phagocyte programmed cell death responses and has inflammatory implications. Epithelial cell apoptotic and autophagic components are mobilized by GAS infection, but can be subverted to ensure bacterial survival. Examination of host interactions with GAS and consequences of GAS infection in the context of cellular receptors responsible for GAS recognition, inflammatory mediator responses, and cell death mechanisms, highlights potential avenues for diagnostic and therapeutic intervention. Understanding the molecular and cellular basis of host symptoms during severe GAS disease will assist the development of improved treatment regimens for this formidable pathogen.

## Introduction

Group A *Streptococcus* (GAS; *Streptococcus pyogenes*) is a clinically important bacterial pathogen responsible for many severe human diseases. Whilst GAS is a frequent agent of self-limited pharyngitis and uncomplicated impetigous infections, penetration of GAS into deeper tissues, trauma, or adverse progression of superficial tissue infections can result in devastating invasive infections, such as the “flesh-eating” syndrome, necrotising fasciitis (NF), or more rarely, myonecrosis [1], [2]. Systemic manifestations of GAS disease, sepsis, or streptococcal toxic shock syndrome (STSS), when accompanying invasive cutaneous infections, strongly contribute to poor patient prognosis [3]. Conservative estimates of the global burden of severe GAS diseases account for 663,000 cases, with 32% of NF cases resulting in patient mortality within seven days in developed countries, rising to a 50% case mortality rate in NF episodes with associated STSS [4], [5]. The destructive nature, rapid onset, and high mortality rate of severe GAS diseases, such as NF and STSS, despite prompt medical care and antimicrobial therapy, necessitates an improved understanding of the mechanisms underlying these pathologies. Whilst mechanisms by which GAS evades elements of the innate immune response have been the focus of intense investigation [6], comparatively less attention has been given to the host processes underlying the pathophysiology of GAS infections. Notably, the roles of cellular host mediators in the context of GAS infection are poorly understood. Here, we integrate recent works describing cellular recognition of GAS infection, signalling pathways leading to inflammatory mediator release and consequent programmed cell death mechanisms. A holistic perspective, incorporating the role of host factors in GAS diseases, should enable the development of more efficacious and tailored treatment options.

## Leukocyte Recognition of GAS and Signalling Pathways

### Toll-like receptor recognition

**Figure 1:**
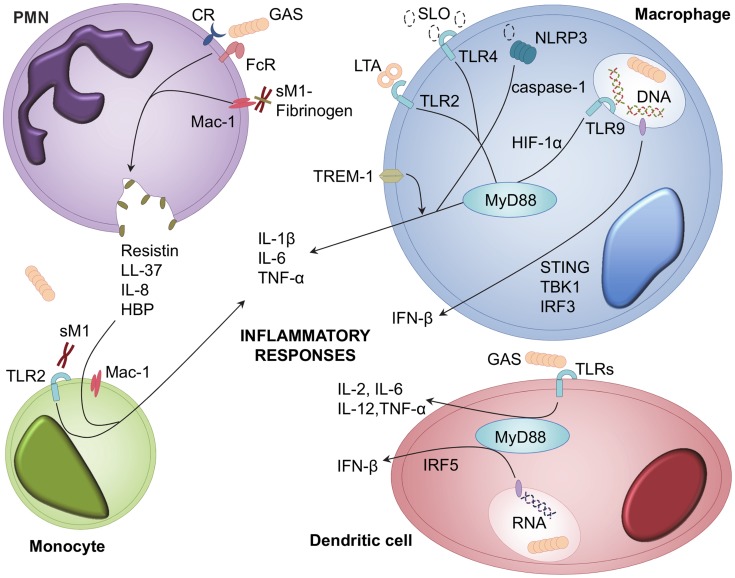
Cellular receptors and signalling pathways involved in GAS recognition and inflammatory mediator release. Inflammatory mediators are released from multiple leukocyte types during GAS infection; including PMNs, monocytes, macrophages, and dendritic cells [12], [13]. GAS and GAS-derived LTA, SLO, and soluble M1 protein (sM1), activate cellular responses to infection [14], [26]. Receptors involved in recognition of GAS include TLRs, TREM-1, complement receptors (CR), immunoglobulin receptors (FcR), Mac-1, and NLRP3 [9], [19], [20], [27]. Ligand binding to these receptors leads to downstream signalling via MyD88, HIF-1α, STING, IFR3, IRF5, and TBK1 [8], [16]–[18]. Recognition of GAS triggers release of interleukins, TNF-α, IFN-β, HBP, resistin, and LL-37 [16], [25].

Cellular surveillance and recognition of foreign agents by the innate immune system is an essential prerequisite to effector recruitment and induction of appropriate responses. One class of cell receptors involved in recognition of highly conserved microbial components are the Toll-like receptors (TLRs). There are currently more than 10 known TLRs, which recognize multiple pathogen-associated molecular patterns (PAMPs), including lipoteichoic acid (LTA), peptidoglycan (TLR2), lipopolysaccharide (TLR4), and unmethylated bacterial CpG DNA (TLR9) [7]. Studies investigating receptors involved in recognition of GAS infection have shown that TLR signalling, particularly through the MyD88 adapter molecule, plays a vital role in induction of host defences and inflammation (Figure 1) [8], [9]. Given that TLR2 recognises multiple gram-positive bacterial ligands, it is likely this TLR plays a major role in GAS recognition and responses. Indeed, induction of streptococcal cell wall extract–induced joint inflammation is dependent on TLR2/MyD88 signalling [10].

### Leukocyte signaling events

Macrophages and dendritic cells (DCs) are commonly viewed as central coordinators of immune responses. This view has been supported by studies showing that macrophages and DCs are both essential for control of GAS infection [11], [12], likely through secretion of multiple pro-inflammatory cytokines such as interleukin (IL)-1β, IL-6, tumour necrosis factor (TNF)-α, and IL-12 [9], [13]. Macrophage and DC recognition of GAS is partially mediated by TLRs, and interaction with multiple bacterial factors by different TLRs appears to provide immune redundancy in inducing cytokine responses. This redundancy is exemplified by the activation of macrophages via TLR4 signalling from nonstructural bacterial elements, such as GAS cytolysin streptolysin O (SLO), and the inability of singular TLR1, TLR2, TLR4, or TLR9 deficiencies to prevent stimulation of DC cytokine production [12], [14]. However, downstream MyD88 signalling is critical for coordinated immune responses, an aspect prominent in clinical studies of patients lacking MyD88, who are highly predisposed to pyogenic infections [15]. MyD88 participates in macrophage and DC TLR-stimulated TNF-α production and type-1 interferon (IFN) responses [8], [9], [16], [17]. GAS induction of IFN-β is nucleic acid–dependent and requires interferon regulator factor (IRF) 3, IRF5, stimulator of interferon genes (STING), and TANK-binding kinase (TBK) 1 signalling via as-yet-undescribed receptors [16], [17]. Although GAS is typically considered an extracellular pathogen [1], recent studies indicate intracellular receptors, such as TLR9, are important for cytokine responses via hypoxia-inducible factor 1-alpha (HIF-1α) signalling, and also contribute to macrophage GAS killing and reactive oxygen species (ROS) production [18].

### Non-TLR recognition of GAS

Other non-TLR receptors have been shown to respond to GAS infection. Secretion of SLO mediates activation of the intracellular NOD-like receptor (NLRP) inflammasome component NLRP3 and promotes macrophage caspase-1 mediated IL-1β secretion independently of both TLR and MyD88 signalling [19]. A recent report describes secondary modulation of macrophage cytokine responses by the triggering receptor expressed on myeloid cells (TREM)-1, which, when inhibited, has a therapeutic effect in murine models of GAS sepsis [20]. The recent work of Baruch et al. demonstrates SLO-mediated host signalling via induction of endoplasmic reticulum stress [21]. Adherence of *sil*-expressing GAS to epithelial cells and macrophages increases host asparagine synthetase expression and asparagine production, driving GAS proliferation and virulence [21].

## Excessive Host Responses Contributing to GAS Disease Pathology

### Superantigenic stimulation of T-cell responses

Sufficient excitation of host defences is necessary to mount an adequate response to combat GAS infection. A large body of evidence suggests, however, that excessive and misdirected host responses underlie damaging pathologies of severe GAS infection [22]. GAS may produce one or more of a large variety of superantigens that increase host propensity to excessive immune activation. Superantigens circumvent conventional antigen presentation by binding simultaneously to both the major histocompatibility complex (MHC) class II and T-cell receptors outside of the peptide-binding area [23]. The resulting “cytokine storm”, driven by activated T-cells and antigen presenting cells, may underlie host hyperinflammatory responses [22]. While GAS superantigens play an important role in dictating immune responses, exhaustive discussion of these protein toxins is beyond the scope of this article, and the reader is directed to recent comprehensive reviews [22]–[24].

### M1-mediated heparin binding protein release

Interactions of polymorphonuclear leukocytes (PMNs) and the classic GAS virulence factor, M protein, have been recently implicated in mediating excessive inflammatory responses [25]. As the major effector cell type of the innate immune system, activation of PMNs and the release of cytotoxic granules has implications for development of severe GAS diseases pathologies. Serotype M1 protein released from the GAS cell surface forms complexes with fibrinogen that bind to PMN Mac-1 receptor, both activating PMNs and triggering release of heparin binding protein (HBP) (Figure 1) [26]. HBP is a potent inducer of vascular permeability, and release of this protein elicits pulmonary lesion formation and vascular leakage [26]. PMN-mediated HBP release synergistically enhances inflammatory cytokine responses from M1 protein–stimulated peripheral blood monocytes during necrotizing infections [27]. Individuals with IgG antibodies directed towards the central region of the M1 protein elicit higher HBP release and are subsequently more susceptible to suffering a pathologically excessive inflammatory response to M1 protein–fibrinogen–IgG complexes [28]. Direct injection of purified M1 protein is sufficient to trigger PMN granule-mediated severe lung damage, which is markedly reduced in neutropenic mice [29]. The M1 protein structure plays an important role in triggering HBP-mediated lung injury, as mutated nonfibrinogen-binding M1 protein exhibits diminished ability to cause pulmonary haemorrhage [30]. The precise organisation of fibrinogen molecules into a supramolecular, cross-like network by M1 protein is essential for PMN activation and is conformationally distinct from normal fibrin clots [31]. A novel marker of septic shock severity, resistin, has also been found to predominately originate from M1 protein–activated PMNs during both systemic and localised severe GAS infection and contributes to local tissue damage [32].

### SLO-mediated inflammatory reactions

Other GAS virulence factors, particularly streptolysin O (SLO), have been found to modulate PMN responses and contribute to inflammation and tissue damage. Oligomerization of SLO in eukaryotic cell membranes forms large (∼25–30 nm) pores and has been shown to disrupt membrane integrity in multiple cell types, including PMNs, macrophages, and epithelial cells [33], [34]. SLO induces toxic PMN platelet aggregates during cutaneous infection that mediate progression of microvascular thrombosis and ischemic tissue necrosis [35]. SLO binding by PMNs has also been shown to mediate HBP, human cathelicidin (LL-37), alpha-defensin, and elastase release [36]. Multiple accounts indicate that SLO secretion also facilitates delivery of other GAS virulence factors. SLO-induced epithelial damage enhanced the penetration of the GAS SpeA superantigen in a vaginal mucosa model ex vivo [37]. SLO production also mediates translocation of GAS NAD-glycohydrolase into host keratinocytes, leading to intracellular signalling [38].

### Host genetic predisposition to excessive immune responses

The role of host genetic factors contributing to GAS disease has been investigated and found to play a decisive role in dictating host susceptibility to severe sepsis and STSS. Goldmann et al. utilised differential susceptibilities of distinct mouse strains to severe GAS infection to demonstrate that failure to control infection and excessive evocation of inflammatory responses by susceptible mice result in extensive tissue destruction [39]. This finding has been refined in studies ascribing differences in severity of STSS to host polymorphism in the human leukocyte antigen complex [13], [40]. Human MHC class II haplotype DRB*1501/DQB1*0602 is less commonly associated with STSS in the presence of NF, while DRB1*07/DQB1*0201 predisposes towards such disease manifestations [40]. A complementary systems genetics approach was recently utilized to identify a panel of host genes bestowing predisposition towards severe GAS sepsis [41]. Pathological levels of the product of one of these genes, Prostaglandin E, directly influences the severity of GAS infection [42].

## Host Leukocyte Cell Death Responses

Programmed cell death plays a decisive role in determining the outcome of microbial infections and inflammation. A variety of regulated cell death mechanisms have been described in response to infection, which have distinct morphological and molecular signatures (Box 1).

**Box 1. Modes of Host Regulated Cell Death**

Multiple pathways leading to host cell death have been described and exhibit characteristic morphological, molecular, and immunological features. Here we briefly summarize key aspects of host regulated cell death mechanisms (reviewed in detail in [78], [79]).

*Apoptosis:* Also known as Type-I cell death, apoptosis is a non-inflammatory cell death mechanism. Apoptosis may be triggered via multiple external (extrinsic) or internal (intrinsic) stimuli. Extrinsic apoptosis follows extracellular signalling via ligands such as Fas/CD95 ligand, TNF-α, and TNF-related apoptosis inducing ligand (TRAIL) to specific death receptors. Intrinsic apoptosis can be induced by various intracellular stresses, including DNA damage and oxidative stress. Morphologically, apoptotic cells undergo cellular shrinkage, nuclear condensation, and blebbing. However, more stringent molecular identification of apoptosis considers dependency upon activation of distinct caspases (typically caspases-3, -6, and -7 for extrinsic apoptosis, and caspases-9 and -3 for intrinsic apoptosis), and the presence of biochemical features such as mitochondrial membrane depolarization, oligonucleosomal DNA fragmentation, ROS generation, exposure of phosphatidylserine, and cellular redistribution of factors such as cytochrome C and Bax. Apoptotic cells are generally phagocytosed by macrophages via efferocytosis; however, if this does not occur, cells may undergo secondary necrosis, whereby intact apoptotic cells lose plasma membrane integrity and undergo autolysis, triggering immune responses.

*Necrosis:* Originally designated “accidental cell death”, necrosis is a proinflammatory cell death mode. While historically regarded as unintentional cell demise in response to overwhelming stress, recent works indicate necrosis may proceed in a regulated fashion (also known as necroptosis or oncosis). Necrosis is exhibited morphologically via cell and organelle swelling, cytoplasmic vacuolization, eventual loss of plasma membrane integrity, and escape of cytoplasmic content, leading to enhanced immune responses. In some instances, regulated necrosis has been linked to necrostatin-inhibitable RIP1-RIP3 activation and/or calpain-cathepsin activation. Most studies investigating programmed necrosis have been conducted using non-infectious disease models (e.g., ischemia-reperfusion); however, a mounting body of evidence suggests a role for this process in infectious disease pathogenesis [80], [81], and warrants further study.

*Autophagic cell death:* Also known as Type-II cell death, autophagic cell death is a non-inflammatory cell death response. Cellular autophagy can be triggered by cells under specific stresses, including endoplasmic reticulum stress or starvation, and are distinguished via the formation of cytoplasmic double-membraned vesicles known as autophagosomes. Autophagosomes typically contain damaged organelles or cytoplasmic material, are labelled with LC3, and eventually fuse with lysosomes (acquiring the LAMP-1 marker), leading to degradation of autophagosomal contents. Autophagic responses that accompany cell death have been associated with a distinct programmed cell death routine; however, an increasing body of evidence supports the supposition that autophagy primarily exerts a protective response against cell demise in vivo.

*Pyroptosis:* Pyroptosis describes a specific form of pro-inflammatory cell death sharing characteristics of both apoptosis and necrosis. Pyroptotic activation of macrophages and other cell types by microbial and nonmicrobial agents is dependent on activation of caspase-1, which proteolytically drives maturation and release of IL-1β and IL-18. Caspase-1 activation is associated with the formation of large multiprotein complexes, either the inflammasome or pyroptosome, which involves signalling via NLRPs and the ASC adaptor protein. These events culminate in cytoplasmic swelling and osmotic lysis of pyroptotic cells, which combined with increased IL-1β and IL-18 release, enhances immune reactions.

### Apoptotic PMN cell death responses

Apoptosis limits the potential of damaged PMNs to ignite inflammatory responses [43]. Coordinated PMN apoptotic shutdown initiates a state of cellular torpor, and administration of apoptotic PMNs actively promotes anti-inflammatory responses [44]. Conversely, necrosis triggers a proinflammatory phenotype resulting in release of damage-associated molecular patterns (DAMPs) and prompts rapid immune responses [45]. In a landmark study by Kobayashi et al., phagocytosis-induced PMN transcriptional responses were analysed for a variety of bacterial pathogens, including GAS [46]. This study provided strong evidence of a common apoptotic program following bacterial uptake and ROS production. PMN apoptotic responses are accelerated in GAS and lead to rapid DNA fragmentation [46]. Although GAS-induced PMN apoptosis was associated with enhanced virulence potential, the virulence determinant(s) responsible for this activity were not elucidated. Later work by the same group indicated PMN apoptosis induced by phagocytosis of latex beads, as opposed to live bacteria, is associated with nullified inflammatory capacity and expedites resolution of inflammation [47].

### GAS cytolysin-induced cell death

Several reports emphasize the dominant role of GAS cytolysins and, to a lesser extent, hyaluronic acid capsule in shaping host cell death. Early studies of SLO and streptolysin S (SLS) function indicate both are capable of inducing plasma membrane permeabilisation, leading to host epithelial cell lysis [33], [48]. GAS strains expressing high and low amounts of SLS were utilised to demonstrate that SLS expression is associated with caspase-7–dependent apoptosis of thioglycolate-induced murine PMNs [49]. However, the strains used in this study also differed significantly in capsule expression, a GAS virulence factor known to influence PMN phagocytosis. Capsule-deficient, GAS-expressing SLO are also more readily phagocytosed by DCs, preventing DC maturation by promoting caspase-dependent apoptosis [50]. Proinflammatory macrophage cell death consistent with regulated necrosis (Box 1) was reported by Goldmann et al. [34]. Macrophage necrosis was attributable to both SLO and SLS activities leading to glycine sensitive pore formation, loss of mitochondrial outer membrane potential (ψ_m_), ROS production, ATP depletion, and calpain activation [34]. Conversely, the work of Timmer et al. [51] found that phagocytosed GAS cytolysin expression also depolarises ψ_m_; however, this leads to DNA fragmentation, cytochrome C redistribution and caspase-3 and -7 dependent apoptotic cell death. Macrophages are principally responsible for the clearance of apoptotic PMNs, and either apoptotic or necrotic depletion of macrophages may exacerbate damage resulting from GAS-induced PMN cell death [52]. Apoptotic PMNs not phagocytosed by macrophages proceed to secondary necrosis and elicitation of inflammatory responses [53]. These data support an emerging theme in bacterial pathogenesis [43], and we propose manipulation of phagocyte cell death responses has inflammatory implications for GAS disease.

### GAS interaction with NETs and NETosis

PMNs are able to release extracellular traps (NETs) composed of DNA, histones, and granule components that ensnare and kill bacteria [54]. GAS and other bacterial pathogens have evolved specialized means of evading this host defence mechanism via secretion of extracellular DNases, which degrade NETs and enable bacterial survival [55]. In vitro studies have indicated PMNs undergo a distinct form of cell death following NET release, dubbed NETosis; however, the relevance of this process to host defence and bacterial pathogenesis has remained contentious [56], [57]. Recent in vivo evidence obtained using intravital microscopy indicates that GAS and other gram-positive pathogens are ensnared by NETosing PMNs; however, these cells, in fact, remain viable and retain bactericidal capacity [58].

## Epithelial Cell Apoptotic Responses

**Figure 2:**
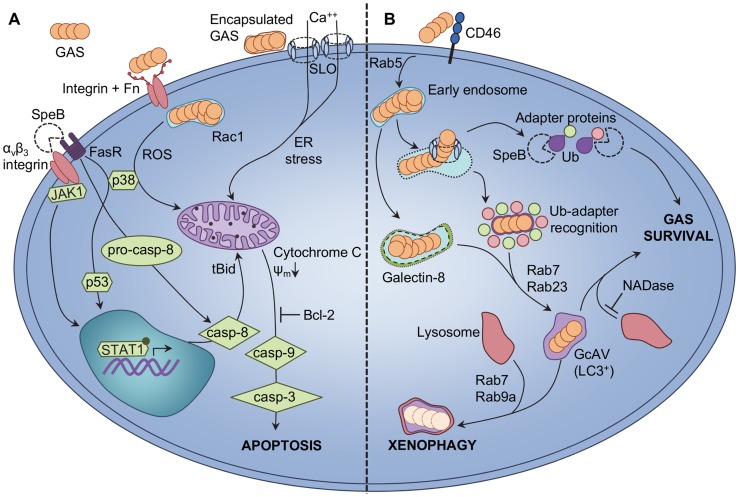
Epithelial cellular responses to GAS infection. **A** GAS-induced apoptosis of epithelial cells is triggered via intrinsic and extrinsic pathways. SpeB binding to epithelial α_v_β_3_ integrins or the Fas receptor (FasR) triggers upregulation of caspase-8 (casp-8) via JAK2, p38, p53, and STAT1 signalling [62]–[64]. Procaspase-8 (pro-casp-8) is also activated directly due to FasR signalling, leading to truncation of cytosolic Bid (tBid) and translocation of tBid to the mitochondria. Extracellular GAS binding to host integrins is enabled via bridging molecules such as fibronectin (Fn), and enables Rac1-mediated internalization of GAS into host epithelial cells. GAS internalization and Rac1 activation facilitates production of ROS, leading to increased p38 phosphorylation [65], [66]. Encapsulated extracellular GAS are not internalized by epithelial cells, and secrete the pore-forming toxin SLO. Integration of SLO into host cell membranes triggers net calcium (Ca^++^) flux into the cytosol and endoplasmic reticulum (ER) stress [67]. All three pathways elicit loss of mitochondrial outer membrane potential (ψ_m_) and release of cytochrome C from the mitochondria, which precedes activation of caspase-9 (casp-9), caspase-3 (casp-3), and apoptosis. Overexpression of the anti-apoptotic factor Bcl-2 by the host can inhibit epithelial cell apoptosis. **B** GAS evades xenophagic killing by epithelial cells. GAS binding to the CD46 receptor is an early signal to activate autophagic responses [72], and GAS are uptaken into early endosomes in a Rab5-dependent manner [74]; however, SLO expression allows GAS to escape from endosomes into the cytosol. GAS exposure to the cytosol is recognized via ubiquitin (Ub) adapter proteins that, in conjunction with Rab7 and Rab23, facilitate shuttling of GAS into GcAVs bearing the classic autophagy LC3 marker [71], [73]. In the absence of SLO, streptolysin S is sufficient to damage endosomal vacuoles for targeting to GcAVs by Ub-independent, galectin-8-mediated autophagy [76]. Lysosomal fusion with GcAVs, via Rab7 and Rab9a, effects xenophagic destruction of intracellular GAS. Expression of SpeB by cytosolic GAS degrades Ub adapter proteins and prevents targeting of bacteria to GcAVs, enhancing intracellular GAS survival [77]. Secretion of GAS NADase also protects GAS from xenophagic killing by inhibiting fusion of GcAVs with lysosomes [76].

Epithelial cells mediate the first interactions between host and bacteria. Adherence of GAS to epithelial receptors frequently precedes intracellular invasion of this bacterium [59], and multiple pathways of epithelial cell death have been reported following GAS infection (Figure 2A). GAS infection of A549 and HEp-2 cells was found to elicit morphological changes consistent with apoptosis, attributable to the activity of GAS cysteine protease SpeB [60]. The apoptotic Fas receptor (FasR) and α_v_β_3_ integrins are able to bind SpeB, leading to downstream caspase-8 activation and translocation of truncated-Bid (tBid) and Bax to mitochondria [61], [62]. Binding of SpeB to host receptors also up-regulates caspase-8 and Bax via JAK-STAT, p38, and p53 signalling [63], [64]. Internalization-dependent epithelial cell apoptosis in response to GAS infection has also been reported, whereby fibronectin-mediated binding of GAS to host integrins triggers actin rearrangement and Rac1 activation [65], [66]. Rac1 activation is hypothesized to mediate epithelial cell ROS production, leading to downstream apoptotic responses [65]–[66]. A third pathway trigged by extracellular GAS also elicits epithelial apoptosis via an SLO-dependent mechanism [67]. Secretion of SLO by encapsulated GAS triggers calcium flux into the cytosol of infected cells, leading to vacuolization of the endoplasmic reticulum and cell apoptosis [67]. Central to these pathways is the role of the mitochondria, as cytochrome c release and ψ_m_ depolarisation are described as key events, and that overexpression of the anti-apoptotic factor Bcl-2 can inhibit mitochondrial dysfunction. In many studies, both capase-9 and caspase-3 are reported to mediate the final apoptotic cascade. Transcriptional analyses of epithelial cells also indicate overall apoptotic responses to GAS infection, and that GAS elicits up-regulation of caspases and calcium regulators [68], [69]. It is important to note that different GAS serotypes do not elicit uniform epithelial responses, and comparison of apoptotic induction by disparate GAS strains demonstrates multiple caspases are utilized [70].

## GAS-Induced Autophagy

Epithelial cell apoptosis has been suggested to protect host cells from infection; however, data validating this hypothesis is lacking, and contention exists regarding whether epithelial apoptotic cell death is, indeed, a protective host response or a pathogenic mechanism utilized by GAS [66], [67]. An alternative epithelial cell defence mechanism against GAS infection has been recently described. Autophagy, a stress response wherein damaged cellular components are targeted to degradative endosomal vacuoles, can protect cells from intracellular GAS (Figure 2B). HeLa cells containing intracellular M6 serotype GAS recognize cytosol-exposed bacteria and target them for autophagic degradation via ubiquitinylation, LC3 labelling, and the formation of GAS-containing, autophagosome-like vacuoles (GcAVs) [71]. Expression of SLO by intracellular GAS is crucial for GAS escape from endosomes into the cytosol and subsequent targeting to GcAVs [71]. Binding of the cell surface CD46 receptor is an early trigger of GAS-mediated autophagy [72]. The Rab-family of G-proteins is responsible for multiple aspects of GcAV formation. Rab7 protein performs numerous roles, including targeting of GAS to GcAVs and initial GcAV formation, and autophagosomal maturation [73]. Other Rab-enzymes, Rab5, Rab23, and Rab9a, also play distinct roles, which include facilitating GAS uptake into endosomes, targeting of GAS to GcAVs, fusion of nascent GcAVs, and autophagosomal maturation [74], [75]. Recent studies report that GAS is able to subvert autophagy-mediated bacterial destruction (also called xenophagy) for intracellular survival. SLO-mediated translocation of GAS NADase prevents efficient killing of serotype M6 GAS via inhibition of autophagosomal fusion with lysosomes [76]. A novel ubiquitin-independent pathway of GcAV formation was also described for primary human keratinocytes in this study, mediated by SLS damage to endosomal vacuoles leading to galectin-8 labelling [76]. Infection of HEp-2 cells with M1T1 serotype GAS reveals significantly greater intracellular survival than by M6 GAS, with M1T1 GAS displaying exposure to the cell cytosol but lack of the LC3 autophagy marker [77]. Enhanced M1T1 GAS survival was found to be directly linked to SpeB activity, which degrades cytosolic ubiquitin and the adapter proteins NDP52, p62, and NBR1 to effect highly efficient evasion of cellular xenophagy [77].

## Discussion

Detailed understanding of the host cellular processes that precipitate severe GAS disease is vital to the development of improved treatment strategies. Host receptor binding to GAS-derived ligands activates PMNs and pro-inflammatory signalling cascades of other cell types, which result in the release of host molecules such as HBP, cytokines and resistin. Excessive exposure of these factors is associated with multiple hallmarks of severe GAS disease, including increased vascular permeability, systemic inflammatory responses and destruction of focal tissues. GAS and GAS cytolysin induction of regulated cell death of leukocytes, leading to increased DAMP release, may further contribute to the virulence and inflammatory potential of this pathogen. While GAS-induced, host leukocyte apoptotic, and regulated necrotic cell death may contribute to GAS disease pathologies, regulated epithelial cell death responses could serve a more protective role, as epithelial xenophagy directly counters GAS intracellular invasion. However, as recent studies illustrate, GAS is able to prevent intracellular killing, and so apoptosis of epithelial cells may serve as a host contingency response, as these cells will be taken up by nearby phagocytes. Thus, therapeutic interventions that reduce the exposure of inflammatory mediators and/or restore native cell death processes may help alleviate pathologies of severe GAS infection.

## References

[1] Walker MJ, Barnett TC, McArthur JD, Cole JN, Gillen CM, et al (2014) Disease manifestations and pathogenic mechanisms of group A *Streptococcus* . Clin Microbiol Rev 27: 264–301. 24696436 10.1128/CMR.00101-13 PMC3993104

[2] Stevens DL, Bisno AL, Chambers HF, Everett ED, Dellinger P, et al (2005) Practice guidelines for the diagnosis and management of skin and soft-tissue infections. Clin Infect Dis 41: 1373–1406. 16231249 10.1086/497143

[3] Stevens DL (2000) Streptococcal toxic shock syndrome associated with necrotizing fasciitis. Annu Rev Med 51: 271–288. 10774464 10.1146/annurev.med.51.1.271

[4] Carapetis JR, Steer AC, Mulholland EK, Weber M (2005) The global burden of group A streptococcal diseases. Lancet Infect Dis 5: 685–694. 16253886 10.1016/S1473-3099(05)70267-X

[5] Lamagni TL, Darenberg J, Luca-Harari B, Siljander T, Efstratiou A, et al (2008) Epidemiology of severe *Streptococcus pyogenes* disease in Europe. J Clin Microbiol 46: 2359–2367. 18463210 10.1128/JCM.00422-08 PMC2446932

[6] Cole JN, Barnett TC, Nizet V, Walker MJ (2011) Molecular insight into invasive group A streptococcal disease. Nat Rev Microbiol 9: 724–736. 21921933 10.1038/nrmicro2648

[7] Takeda K, Kaisho T, Akira S (2003) Toll-like receptors. Annu Rev Immunol 21: 335–376. 12524386 10.1146/annurev.immunol.21.120601.141126

[8] Loof TG, Goldmann O, Gessner A, Herwald H, Medina E (2010) Aberrant inflammatory response to *Streptococcus pyogenes* in mice lacking myeloid differentiation factor 88. Am J Pathol 176: 754–763. 20019195 10.2353/ajpath.2010.090422 PMC2808082

[9] Loof TG, Goldmann O, Medina E (2008) Immune recognition of *Streptococcus pyogenes* by dendritic cells. Infect Immun 76: 2785–2792. 18391010 10.1128/IAI.01680-07 PMC2423069

[10] Joosten LA, Koenders MI, Smeets RL, Heuvelmans-Jacobs M, Helsen MM, et al (2003) Toll-like receptor 2 pathway drives streptococcal cell wall-induced joint inflammation: critical role of myeloid differentiation factor 88. J Immunol 171: 6145–6153. 14634130 10.4049/jimmunol.171.11.6145

[11] Goldmann O, Rohde M, Chhatwal GS, Medina E (2004) Role of macrophages in host resistance to group A streptococci. Infect Immun 72: 2956–2963. 15102808 10.1128/IAI.72.5.2956-2963.2004 PMC387899

[12] Loof TG, Rohde M, Chhatwal GS, Jung S, Medina E (2007) The contribution of dendritic cells to host defenses against *Streptococcus pyogenes* . J Infect Dis 196: 1794–1803. 18190260 10.1086/523647

[13] Goldmann O, Lengeling A, Bose J, Bloecker H, Geffers R, et al (2005) The role of the MHC on resistance to group A streptococci in mice. J Immunol 175: 3862–3872. 16148132 10.4049/jimmunol.175.6.3862

[14] Park JM, Ng VH, Maeda S, Rest RF, Karin M (2004) Anthrolysin O and other Gram-positive cytolysins are toll-like receptor 4 agonists. J Exp Med 200: 1647–1655. 15611291 10.1084/jem.20041215 PMC2211988

[15] von Bernuth H, Picard C, Jin Z, Pankla R, Xiao H, et al (2008) Pyogenic bacterial infections in humans with MyD88 deficiency. Science 321: 691–696. 18669862 10.1126/science.1158298 PMC2688396

[16] Gratz N, Siller M, Schaljo B, Pirzada ZA, Gattermeier I, et al (2008) Group A *Streptococcus* activates type I interferon production and MyD88-dependent signaling without involvement of TLR2, TLR4, and TLR9. J Biol Chem 283: 19879–19887. 18480050 10.1074/jbc.M802848200 PMC2459277

[17] Gratz N, Hartweger H, Matt U, Kratochvill F, Janos M, et al (2011) Type I interferon production induced by *Streptococcus pyogenes*-derived nucleic acids is required for host protection. PLoS Pathog 7: e1001345. 21625574 10.1371/journal.ppat.1001345 PMC3098218

[18] Zinkernagel AS, Hruz P, Uchiyama S, von Kockritz-Blickwede M, Schuepbach RA, et al (2012) Importance of Toll-like receptor 9 in host defense against M1T1 group A *Streptococcus* infections. J Innate Immun 4: 213–218. 21860217 10.1159/000329550 PMC3388268

[19] Harder J, Franchi L, Munoz-Planillo R, Park JH, Reimer T, et al (2009) Activation of the Nlrp3 inflammasome by *Streptococcus pyogenes* requires streptolysin O and NF-kappa B activation but proceeds independently of TLR signaling and P2X7 receptor. J Immunol 183: 5823–5829. 19812205 10.4049/jimmunol.0900444 PMC2765568

[20] Horst SA, Linner A, Beineke A, Lehne S, Holtje C, et al (2013) Prognostic value and therapeutic potential of TREM-1 in *Streptococcus pyogenes*-induced sepsis. J Innate Immun 5: 581–590. 23571837 10.1159/000348283 PMC6741602

[21] Baruch M, Belotserkovsky I, Hertzog BB, Ravins M, Dov E, et al (2014) An extracellular bacterial pathogen modulates host metabolism to regulate its own sensing and proliferation. Cell 156: 97–108. 24439371 10.1016/j.cell.2013.12.007 PMC3926133

[22] Lappin E, Ferguson AJ (2009) Gram-positive toxic shock syndromes. Lancet Infect Dis 9: 281–290. 19393958 10.1016/S1473-3099(09)70066-0

[23] Fraser JD, Proft T (2008) The bacterial superantigen and superantigen-like proteins. Immunol Rev 225: 226–243. 18837785 10.1111/j.1600-065X.2008.00681.x

[24] Commons RJ, Smeesters PR, Proft T, Fraser JD, Robins-Browne R, et al (2014) Streptococcal superantigens: categorization and clinical associations. Trends Mol Med 20: 48–62. 24210845 10.1016/j.molmed.2013.10.004

[25] Norrby-Teglund A, Johansson L (2013) Beyond the traditional immune response: bacterial interaction with phagocytic cells. Int J Antimicrob Agents 42: 13–16. 10.1016/j.ijantimicag.2013.04.005 23684004

[26] Herwald H, Cramer H, Morgelin M, Russell W, Sollenberg U, et al (2004) M protein, a classical bacterial virulence determinant, forms complexes with fibrinogen that induce vascular leakage. Cell 116: 367–379. 15016372 10.1016/s0092-8674(04)00057-1

[27] Pahlman LI, Morgelin M, Eckert J, Johansson L, Russell W, et al (2006) Streptococcal M protein: a multipotent and powerful inducer of inflammation. J Immunol 177: 1221–1228. 16818781 10.4049/jimmunol.177.2.1221

[28] Kahn F, Morgelin M, Shannon O, Norrby-Teglund A, Herwald H, et al (2008) Antibodies against a surface protein of *Streptococcus pyogenes* promote a pathological inflammatory response. PLoS Pathog 4: e1000149. 18787689 10.1371/journal.ppat.1000149 PMC2522270

[29] Soehnlein O, Oehmcke S, Ma X, Rothfuchs AG, Frithiof R, et al (2008) Neutrophil degranulation mediates severe lung damage triggered by streptococcal M1 protein. Eur Respir J 32: 405–412. 18321926 10.1183/09031936.00173207

[30] McNamara C, Zinkernagel AS, Macheboeuf P, Cunningham MW, Nizet V, et al (2008) Coiled-coil irregularities and instabilities in group A *Streptococcus* M1 are required for virulence. Science 319: 1405–1408. 18323455 10.1126/science.1154470 PMC2288698

[31] Macheboeuf P, Buffalo C, Fu CY, Zinkernagel AS, Cole JN, et al (2011) Streptococcal M1 protein constructs a pathological host fibrinogen network. Nature 472: 64–68. 21475196 10.1038/nature09967 PMC3268815

[32] Johansson L, Linner A, Sunden-Cullberg J, Haggar A, Herwald H, et al (2009) Neutrophil-derived hyperresistinemia in severe acute streptococcal infections. J Immunol 183: 4047–4054. 19717514 10.4049/jimmunol.0901541

[33] Sierig G, Cywes C, Wessels MR, Ashbaugh CD (2003) Cytotoxic effects of streptolysin O and streptolysin S enhance the virulence of poorly encapsulated group A streptococci. Infect Immun 71: 446–455. 12496195 10.1128/IAI.71.1.446-455.2003 PMC143243

[34] Goldmann O, Sastalla I, Wos-Oxley M, Rohde M, Medina E (2009) *Streptococcus pyogenes* induces oncosis in macrophages through the activation of an inflammatory programmed cell death pathway. Cell Microbiol 11: 138–155. 19016794 10.1111/j.1462-5822.2008.01245.x

[35] Bryant AE, Bayer CR, Chen RY, Guth PH, Wallace RJ, et al (2005) Vascular dysfunction and ischemic destruction of tissue in *Streptococcus pyogenes* infection: the role of streptolysin O-induced platelet/neutrophil complexes. J Infect Dis 192: 1014–1022. 16107954 10.1086/432729

[36] Nilsson M, Sorensen OE, Morgelin M, Weineisen M, Sjobring U, et al (2006) Activation of human polymorphonuclear neutrophils by streptolysin O from *Streptococcus pyogenes* leads to the release of proinflammatory mediators. Thromb Haemost 95: 982–990. 16732377 10.1160/TH05-08-0572

[37] Brosnahan AJ, Mantz MJ, Squier CA, Peterson ML, Schlievert PM (2009) Cytolysins augment superantigen penetration of stratified mucosa. J Immunol 182: 2364–2373. 19201891 10.4049/jimmunol.0803283 PMC2805182

[38] Bricker AL, Cywes C, Ashbaugh CD, Wessels MR (2002) NAD(+)-glycohydrolase acts as an intracellular toxin to enhance the extracellular survival of group A streptococci. Mol Microbiol 44: 257–269. 11967084 10.1046/j.1365-2958.2002.02876.x

[39] Goldmann O, Chhatwal GS, Medina E (2003) Immune mechanisms underlying host susceptibility to infection with group A streptococci. J Infect Dis 187: 854–861. 12599060 10.1086/368390

[40] Kotb M, Norrby-Teglund A, McGeer A, El-Sherbini H, Dorak MT, et al (2002) An immunogenetic and molecular basis for differences in outcomes of invasive group A streptococcal infections. Nat Med 8: 1398–1404. 12436116 10.1038/nm1202-800

[41] Abdeltawab NF, Aziz RK, Kansal R, Rowe SL, Su Y, et al (2008) An unbiased systems genetics approach to mapping genetic loci modulating susceptibility to severe streptococcal sepsis. PLoS Pathog 4: e1000042. 18421376 10.1371/journal.ppat.1000042 PMC2277464

[42] Goldmann O, Hertzen E, Hecht A, Schmidt H, Lehne S, et al (2010) Inducible cyclooxygenase released prostaglandin E2 modulates the severity of infection caused by *Streptococcus pyogenes* . J Immunol 185: 2372–2381. 20644176 10.4049/jimmunol.1000838

[43] Kennedy AD, Deleo FR (2009) Neutrophil apoptosis and the resolution of infection. Immunol Res 43: 25–61. 19066741 10.1007/s12026-008-8049-6

[44] Ren Y, Xie Y, Jiang G, Fan J, Yeung J, et al (2008) Apoptotic cells protect mice against lipopolysaccharide-induced shock. J Immunol 180: 4978–4985. 18354223 10.4049/jimmunol.180.7.4978

[45] Kono H, Rock KL (2008) How dying cells alert the immune system to danger. Nat Rev Immunol 8: 279–289. 18340345 10.1038/nri2215 PMC2763408

[46] Kobayashi SD, Braughton KR, Whitney AR, Voyich JM, Schwan TG, et al (2003) Bacterial pathogens modulate an apoptosis differentiation program in human neutrophils. Proc Natl Acad Sci U S A 100: 10948–10953. 12960399 10.1073/pnas.1833375100 PMC196908

[47] Kobayashi SD, Voyich JM, Braughton KR, DeLeo FR (2003) Down-regulation of proinflammatory capacity during apoptosis in human polymorphonuclear leukocytes. J Immunol 170: 3357–3368. 12626596 10.4049/jimmunol.170.6.3357

[48] Datta V, Myskowski SM, Kwinn LA, Chiem DN, Varki N, et al (2005) Mutational analysis of the group A streptococcal operon encoding streptolysin S and its virulence role in invasive infection. Mol Microbiol 56: 681–695. 15819624 10.1111/j.1365-2958.2005.04583.x

[49] Miyoshi-Akiyama T, Takamatsu D, Koyanagi M, Zhao JZ, Imanishi K, et al (2005) Cytocidal effect of *Streptococcus pyogenes* on mouse neutrophils in vivo and the critical role of streptolysin S. J Infect Dis 192: 107–116. 15942900 10.1086/430617

[50] Cortes G, Wessels MR (2009) Inhibition of dendritic cell maturation by group A *Streptococcus* . J Infect Dis 200: 1152–1161. 19712038 10.1086/605696 PMC2845989

[51] Timmer AM, Timmer JC, Pence MA, Hsu LC, Ghochani M, et al (2009) Streptolysin O promotes group A *Streptococcus* immune evasion by accelerated macrophage apoptosis. J Biol Chem 284: 862–871. 19001420 10.1074/jbc.M804632200 PMC2613605

[52] Silva MT (2011) Macrophage phagocytosis of neutrophils at inflammatory/infectious foci: a cooperative mechanism in the control of infection and infectious inflammation. J Leukoc Biol 89: 675–683. 21169518 10.1189/jlb.0910536

[53] Silva MT (2010) Bacteria-induced phagocyte secondary necrosis as a pathogenicity mechanism. J Leukoc Biol 88: 885–896. 20566623 10.1189/jlb.0410205

[54] Brinkmann V, Reichard U, Goosmann C, Fauler B, Uhlemann Y, et al (2004) Neutrophil extracellular traps kill bacteria. Science 303: 1532–1535. 15001782 10.1126/science.1092385

[55] Buchanan JT, Simpson AJ, Aziz RK, Liu GY, Kristian SA, et al (2006) DNase expression allows the pathogen group A *Streptococcus* to escape killing in neutrophil extracellular traps. Curr Biol 16: 396–400. 16488874 10.1016/j.cub.2005.12.039

[56] Fuchs TA, Abed U, Goosmann C, Hurwitz R, Schulze I, et al (2007) Novel cell death program leads to neutrophil extracellular traps. J Cell Biol 176: 231–241. 17210947 10.1083/jcb.200606027 PMC2063942

[57] Lu T, Kobayashi SD, Quinn MT, Deleo FR (2012) A NET outcome. Front Immunol 3: 365. 23227026 10.3389/fimmu.2012.00365 PMC3514450

[58] Yipp BG, Petri B, Salina D, Jenne CN, Scott BN, et al (2012) Infection-induced NETosis is a dynamic process involving neutrophil multitasking in vivo. Nat Med 18: 1386–1393. 22922410 10.1038/nm.2847 PMC4529131

[59] Nitsche-Schmitz DP, Rohde M, Chhatwal GS (2007) Invasion mechanisms of Gram-positive pathogenic cocci. Thromb Haemost 98: 488–496. 17849036

[60] Tsai PJ, Lin YS, Kuo CF, Lei HY, Wu JJ (1999) Group A *Streptococcus* induces apoptosis in human epithelial cells. Infect Immun 67: 4334–4339. 10456871 10.1128/iai.67.9.4334-4339.1999 PMC96749

[61] Tsai WH, Chang CW, Chuang WJ, Lin YS, Wu JJ, et al (2004) Streptococcal pyrogenic exotoxin B-induced apoptosis in A549 cells is mediated by a receptor- and mitochondrion-dependent pathway. Infect Immun 72: 7055–7062. 15557629 10.1128/IAI.72.12.7055-7062.2004 PMC529174

[62] Tsai WH, Chang CW, Lin YS, Chuang WJ, Wu JJ, et al (2008) Streptococcal pyrogenic exotoxin B-induced apoptosis in A549 cells is mediated through alpha(v)beta(3) integrin and Fas. Infect Immun 76: 1349–1357. 18227168 10.1128/IAI.01162-07 PMC2292884

[63] Chang CW, Tsai WH, Chuang WJ, Lin YS, Wu JJ, et al (2009) Procaspase 8 and Bax are up-regulated by distinct pathways in streptococcal pyrogenic exotoxin B-induced apoptosis. J Biol Chem 284: 33195–33205. 19801665 10.1074/jbc.M109.020586 PMC2785162

[64] Lee WT, Chang CW (2010) Bax is upregulated by p53 signal pathway in the SPE B-induced apoptosis. Mol Cellular Biochem 343: 271–279. 20567883 10.1007/s11010-010-0522-6

[65] Nakagawa I, Nakata M, Kawabata S, Hamada S (2001) Cytochrome c-mediated caspase-9 activation triggers apoptosis in *Streptococcus pyogenes*-infected epithelial cells. Cell Microbiol 3: 395–405. 11422082 10.1046/j.1462-5822.2001.00122.x

[66] Aikawa C, Nozawa T, Maruyama F, Tsumoto K, Hamada S, et al (2010) Reactive oxygen species induced by *Streptococcus pyogenes* invasion trigger apoptotic cell death in infected epithelial cells. Cell Microbiol 12: 814–830. 20070306 10.1111/j.1462-5822.2010.01435.x

[67] Cywes Bentley C, Hakansson A, Christianson J, Wessels MR (2005) Extracellular group A *Streptococcus* induces keratinocyte apoptosis by dysregulating calcium signalling. Cell Microbiol 7: 945–955. 15953027 10.1111/j.1462-5822.2005.00525.x

[68] Klenk M, Koczan D, Guthke R, Nakata M, Thiesen HJ, et al (2005) Global epithelial cell transcriptional responses reveal *Streptococcus pyogenes* Fas regulator activity association with bacterial aggressiveness. Cell Microbiol 7: 1237–1250. 16098212 10.1111/j.1462-5822.2005.00548.x

[69] Nakagawa I, Nakata M, Kawabata S, Hamada S (2004) Transcriptome analysis and gene expression profiles of early apoptosis-related genes in *Streptococcus pyogenes*-infected epithelial cells. Cell Microbiol 6: 939–952. 15339269 10.1111/j.1462-5822.2004.00412.x

[70] Klenk M, Nakata M, Podbielski A, Skupin B, Schroten H, et al (2007) *Streptococcus pyogenes* serotype-dependent and independent changes in infected HEp-2 epithelial cells. ISME J 1: 678–692. 18059492 10.1038/ismej.2007.54

[71] Nakagawa I, Amano A, Mizushima N, Yamamoto A, Yamaguchi H, et al (2004) Autophagy defends cells against invading group A *Streptococcus* . Science 306: 1037–1040. 15528445 10.1126/science.1103966

[72] Joubert PE, Meiffren G, Gregoire IP, Pontini G, Richetta C, et al (2009) Autophagy induction by the pathogen receptor CD46. Cell Host Microbe 6: 354–366. 19837375 10.1016/j.chom.2009.09.006

[73] Yamaguchi H, Nakagawa I, Yamamoto A, Amano A, Noda T, et al (2009) An initial step of GAS-containing autophagosome-like vacuoles formation requires Rab7. PLoS Pathog 5: e1000670. 19956673 10.1371/journal.ppat.1000670 PMC2777386

[74] Sakurai A, Maruyama F, Funao J, Nozawa T, Aikawa C, et al (2010) Specific behavior of intracellular *Streptococcus pyogenes* that has undergone autophagic degradation is associated with bacterial streptolysin O and host small G proteins Rab5 and Rab7. J Biol Chem 285: 22666–22675. 20472552 10.1074/jbc.M109.100131 PMC2903418

[75] Nozawa T, Aikawa C, Goda A, Maruyama F, Hamada S, et al (2012) The small GTPases Rab9A and Rab23 function at distinct steps in autophagy during group A *Streptococcus* infection. Cell Microbiol 14: 1149–1165. 22452336 10.1111/j.1462-5822.2012.01792.x

[76] O'Seaghdha M, Wessels MR (2013) Streptolysin O and its co-toxin NAD-glycohydrolase protect group A *Streptococcus* from xenophagic killing. PLoS Pathog 9: e1003394. 23762025 10.1371/journal.ppat.1003394 PMC3675196

[77] Barnett TC, Liebl D, Seymour LM, Gillen CM, Lim JY, et al (2013) The globally disseminated M1T1 clone of group A *Streptococcus* evades autophagy for intracellular replication. Cell Host Microbe 14: 675–682. 24331465 10.1016/j.chom.2013.11.003 PMC3918495

[78] Kroemer G, Galluzzi L, Vandenabeele P, Abrams J, Alnemri ES, et al (2009) Classification of cell death: recommendations of the Nomenclature Committee on Cell Death 2009. Cell Death Diff 16: 3–11. 10.1038/cdd.2008.150 PMC2744427 18846107

[79] Galluzzi L, Vitale I, Abrams JM, Alnemri ES, Baehrecke EH, et al (2012) Molecular definitions of cell death subroutines: recommendations of the Nomenclature Committee on Cell Death 2012. Cell Death Diff 19: 107–120. 10.1038/cdd.2011.96 PMC3252826 21760595

[80] Robinson N, McComb S, Mulligan R, Dudani R, Krishnan L, et al (2012) Type I interferon induces necroptosis in macrophages during infection with *Salmonella enterica* serovar *Typhimurium* . Nat Immunol 13: 954–962. 22922364 10.1038/ni.2397 PMC4005791

[81] Cho Y, Challa S, Moquin D, Genga R, Ray TD, et al (2009) Phosphorylation-driven assembly of the RIP1-RIP3 complex regulates programmed necrosis and virus-induced inflammation. Cell 137: 1112–1123. 19524513 10.1016/j.cell.2009.05.037 PMC2727676

